# Complete Genome Sequence of Foot-and-Mouth Disease Virus Serotype O Strain YNTBa from Yunnan Province of China

**DOI:** 10.1128/genomeA.00393-17

**Published:** 2017-06-01

**Authors:** Huan Yan, Jihua Wang, Mingwang Zhu, Haisheng Miao, Zhenqi Peng, Huachun Li, Aiguo Xin

**Affiliations:** a Yunnan Animal Science and Veterinary Institute, Kunming, Yunnan, China; b Yunnan Provincial Research Center for Veterinary Biological Products, Baoshan, Yunnan, China; c Faculty of Animal Science and Technology, Yunnan Agricultural University, Kunming, Yunnan, China

## Abstract

YNTBa is a rabbit-passaged attenuated strain of foot-and-mouth disease virus (FMDV) serotype O. Here, we announce the complete genome sequence of YNTBa, which provides data for further studies on replication, virulence, its determinants, and cell and host tropism of YNTBa.

Foot-and-mouth disease virus (FMDV), a member of the genus *Aphthovirus* within the family *Picornaviridae*, contains a single-stranded positive-sense RNA genome of approximately 8.3 kb, surrounded by an icosahedral capsid comprising four different structural proteins (VP1 to VP4, 60 copies for each) (1, 2). FMDV can be divided into seven serotypes: O, A, C, Asia1, and Southern African Territories 1 to 3 (SAT1 to -3) (1, 2). Among the seven serotypes of FMDV, serotype O shows the broadest distribution worldwide (3). In the late 1950s, an outbreak of FMDV was first detected in Yunnan Province of China. An FMDV strain was isolated from skin vesicles in cattle and named as the YNTB (Yunnan TaiBao) strain. Subsequently, the researchers took initiative to develop live-attenuated vaccines to control the FMD outbreaks on the border between China and Myanmar. The highly virulent YNTB was serially passaged in suckling rabbits more than 400 times, with the viral virulence markedly reduced in yellow cattle but still causing clinical symptoms in buffalo.

Here, we report the complete genome sequence of the YNTB attenuation (YNTBa) strain. YNTBa was derived from a virulent parental YNTB strain via consecutive passage 480 times in suckling rabbits and then was adapted in BHK-21 cells. Viral RNA was extracted from the viral supernatant of BHK-21 cells, and cDNA was synthesized using an oligo(dT) primer or gene-specific primers. Seven PCRs were performed to obtain overlapping sequences which covered the entire YNTBa genome. PCR products were subjected to direct sequencing and assembled using the software ContigExpress (Vector NTI Advance 11; Invitrogen). Phylogenetic analysis was performed using the MEGA 6.0 software.

The complete genome of YNTBa is 8,176 nucleotides (nt) in length, including a 1,055-nt 5′ untranslated region (5′ UTR) with 18 nt of a poly(C) tract and a 122-nt 3′ UTR with a 30-nt poly(A) tail. A 6,999-nt single open reading frame (ORF) was predicted to encode a polyprotein of 2,332 amino acids (aa) containing four structural and eight nonstructural proteins. YNTBa belongs to the Cathay topotype of FMDV serotype O. YNTBa shared the highest identities (99%) with the Akesu58 and OMIII strains isolated from China. A comparison of YNTBa with its virulent parental strain revealed that no deletion/insertion mutations occurred. In the protein-coding region, a total 48 amino acid mutations were observed to be scattered across 10 proteins. The L^pro^, VP2, VP1, 2B, and 2C proteins were found to be the most frequent mutational regions. The VP4, VP3, 3A, 3B, and 3D^pol^ had lower mutation frequencies, and no mutations were found in 2A or 3C.

Historically, FMD attenuation had been achieved by passaging in alternate hosts and/or in tissue culture (4). The criteria for the selection of the FMDV attenuation strain were highly empirical, and little is known about the molecular mechanisms underlying attenuation (5). The complete genetic information of YNTBa will be helpful for further elucidation of the relationship between host recognition and virulence.

## Accession number(s). 

The complete genome sequence of a virulent attenuated strain (YNTBa) has been submitted to GenBank under the accession number KY072818.
